# Dr. Frances Weidman-Hynds

**Published:** 1896-02

**Authors:** 


Dr. Frances Weidman-Hynds, of Brooklyn, died at her residence, 807 Union avenue, in that city, December 5, 1895, after a short illness. She was a graduate of Buffalo University medical college, class of '91, and was for three years afterward resident physician of the Englewood hospital at Englewood, N. J.

Her classmates and many friends in Buffalo will deeply regret to learn of her demise.

